# Involvement of COX-2/PGE_**2**_ Pathway in the Upregulation of MMP-9 Expression in Pancreatic Cancer

**DOI:** 10.1155/2011/214269

**Published:** 2011-06-29

**Authors:** Xianmin Bu, Chenghai Zhao, Xianwei Dai

**Affiliations:** ^1^Department of General Surgery, Shengjing Hospital, China Medical University, Shenyang 110004, China; ^2^Department of Pathophysiology, College of Basic Medical Science, China Medical University, Shenyang 110001, China

## Abstract

COX-2 and MMP-9 have been reported to show an overexpression in pancreatic cancer, and thus an attempt to explore the correlation between them has become a target of this study. Besides, PGE_2_, a product of COX-2, was also under research as to whether it is involved in the upregulation of MMP-9 expression by COX-2. Expression of COX-2 and MMP-9 mRNA varied in pancreatic adenocarcinomas, and the mRNA level of COX-2 was correlated positively with MMP-9. Both BxPC-3 and Capan-1 cells had strong expression of COX-2 and MMP-9. MMP-9 expression was downregulated significantly in BxPC-3 and Capan-1 cells after treatment with COX-2 inhibitors or COX-2 siRNA plasmids, and upregulated in BxPC-3 significantly by exogenous TNF-*α*, LPS or PGE_2_. The upregulation of MMP-9 by TNF-*α* or LPS was inhibited by COX-2 inhibitor NS398. There was a significant increase in the migration of BxPC-3 cells with TNF-*α*, LPS, or PGE_2_ treatment; however, the increase caused by TNF-*α* or LPS was also inhibited remarkably by NS398. Our findings demonstrated that COX-2 upregulates MMP-9 expression in pancreatic cancer, and PGE_2_ may be involved in it.

## 1. Introduction

Overexpression of cyclooxygenase-2 (COX-2) has been found in a series of human cancers [1–7], which suggests its linkage to the development of these tumours. Specifically, its action in carcinogenesis is generally thought to be mediated by COX-2-generated prostanoids, especially prostaglandin E_2_ (PGE_2_), the most abundant prostanoid in human body, by stimulating cell proliferation, invasion, and angiogenesis [8–13]. Several other studies demonstrate an overexpression of COX-2 in pancreatic cancer [14–16] and a suppression of COX-2 inhibitors on the proliferation [17].

Pancreatic cancer, one of the most lethal malignancies, is usually diagnosed when perineural invasion and distal metastasis are already present. However, mechanisms of the initial phase are so far not completely understood. Though the correlation of COX-2 expression with perineural invasion in pancreatic cancer patients has been generally examined [18], the exact process remains unclear and still needs to pin down.

Matrix metalloproteinases (MMPs), as zinc containing enzymes, are labelled the agent to degrade extracellular matrix components and to play a crucial role in invasion and metastasis of cancer cells. MMP-9 from the family is constantly upregulated in a variety of human cancers, and this has aroused our interest to investigate the correlation of COX-2 with MMP-9 in pancreatic cancer. Likewise, the involvement of PGE_2_ (as a product of COX-2) in the upregulation of MMP-9 expression by COX-2 was also under research.

## 2. Methods

### 2.1. Pancreatic Cancer Cell Lines and Tissue Specimens

The human pancreatic cancer cell lines BxPC-3, Capan-1, PANC-1, and AsPC-1 were obtained from ATCC (Rockville, MD, USA) and were cultured in RPMI 1640 supplemented with 10% fetal bovine serum, at 37°C in a humid incubator with 5% CO_2_. 16 pancreatic adenocarcinoma specimens were acquired from patients under operation with all their informed consent at Shengjing hospital, Chinese Medical University and were frozen in liquid nitrogen immediately after surgical removal. This study was carried out with the approval of the ethical committee of China Medical University. 

### 2.2. RT-PCR and Real-Time PCR

Total RNA was isolated from tissues and cell lines by Trizol (Takara, Dalian, China) according to the protocol supplied by the manufacturers. Then, 1 *μ*g of RNA was used to synthesize first-strand cDNA. RT-PCR was carried out by Takara RNA PCR 3.0 Kit (Takara, Dalian, China). Real-time PCR was performed using the LightCycler system together with the LightCycler DNA Master SYBR Green I Kit (Roche Diagnostics). The housekeeping gene glyceraldehyde-3-phosphate dehydrogenase (GAPDH) was used as an internal control. Gene expression was quantified by the comparative CT method, normalizing CT values to GAPDH and calculating relative expression values. Primer sequences were as follows: COX-2 forward, 5′-ACAATGCTGACTATGGCTAC-3′, reverse, 5′-CTGATGCGTGAAGTGCTG-3′; MMP-9 forward, 5′-AGGACGGCAATGCTGATG-3′, reverse, 5′-TCGTAGTTGGCGGTGGTG-3′; GAPDH forward, 5′-GGGAAACTGTGGCGTGAT-3′, reverse, 5′-AAAGGTGGAGGAGTGGGT-3′.

### 2.3. Western Blotting

Cell lysates were prepared with sample buffer (50 mmol/L Tris-HCl (pH 6.8), 100 mmol/L DTT, 2% SDS, 0.1% bromophenol blue, and 10% glycerol) and were subjected to a 12% sodium dodecyl sulfate (SDS)/acrylamide gel. The proteins on acrylamide gel were transferred to a nylon membrane, which was blocked overnight (4°C in PBS with 0.1% Tween and 10% milk powder). Primary antibodies for COX-2 and MMP-9 (Santa Cruz, CA, America), and the corresponding secondary antibodies (Santa Cruz, CA, America) were applied before immunoblotting. The human gene *β-actin *(Santa Cruz, CA, America) was used as an internal control. Blots were visualized with FX pro plus system (Bio-Rad) and quantified using Scion Image 4.03 software. 

### 2.4. RNA Interference

COX-2 siRNA plasmid and nonsilencing control siRNA plasmid were purchased from Takala (Dalian, China). Cells were seeded into a 24-well plate at a density of 2 × 10^5^. On the following day, cells were transfected with COX-2 siRNA or control siRNA using Lipofectamine 2000 (Invitrogen, United Kingdom) according to the manufacturer's instructions. 

### 2.5. Elisa for PGE_2_ in Cell Culture Supernatants

Concentrations of PGE_2_ in cell culture supernatants were measured using the Quantikine Elisa kit (Boster, Wuhan, China) according to the manufacturer's instructions. The sensitivity of the assay was 2 pg/mL.

### 2.6. Migration Assays

The migration of cultured cells was assayed using Matrigel invasion chamber (24-well format, 8 *μ*m pore; BD pharmingen). Cells (5 × 10^5^) were added to the upper chamber. After 24 hours at 37°C and 5% CO_2_, migrated cells on the lower surface were stained using 1% toluidine blue after fixation with 100% methanol. For each transwell, the number of migrated cells in 10 fields (×200) was counted.

### 2.7. Statistical Analysis

Correlation between COX-2 expression and MMP-9 expression in pancreatic cancer specimens was analyzed using Spearman's rank correlation test. Expression of mRNA was compared by Student's* t*-test in pancreatic cell lines. Statistical analysis was carried out using SPSS version 11.0 (SPSS, Chicago, IL, USA). Difference was considered significant when *P*-value was <0.05.

## 3. Results

### 3.1. Expression of COX-2 and MMP-9 mRNA in Pancreatic Cancers

**Figure 1:**
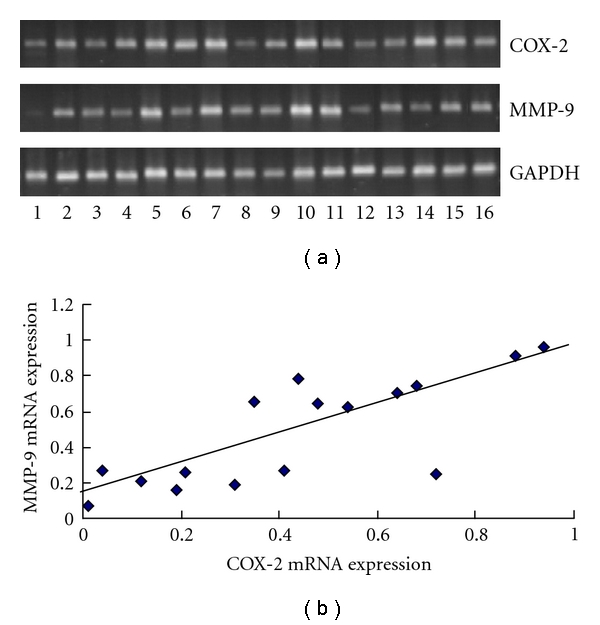
Expression of COX-2 and MMP-9 mRNA in pancreatic cancers. (a) Expression of COX-2 and MMP-9 mRNA was detected in 16 pancreatic cancers by RT-PCR. (b) Level of MMP-9 mRNA was correlated positively with that of COX-2 mRNA in 16 pancreatic cancers by real-time PCR, *P* < 0.01.

COX-2 and MMP-9 mRNA were initially detected in 16 pancreatic adenocarcinomas by RT-PCR, and then their expression was found to have varied in these cancers (Figure 1). In an attempt to evaluate the presumed correlation of COX-2 mRNA expression with MMP-9, we further determined their mRNA level using real-time PCR, and as hypothesized, Spearman's rank correlation test verified a positive one in overall 16 pancreatic cancers (*P* < 0.01, Figure 1).

### 3.2. Differential Expression of COX-2 and MMP-9 in Pancreatic Cancer Cell Lines

**Figure 2:**
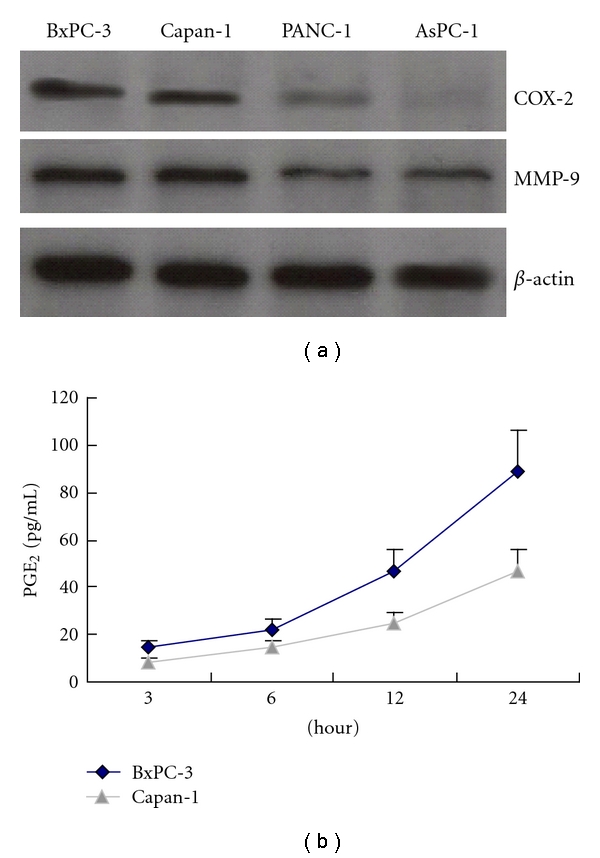
Expression of COX-2 and MMP-9 in pancreatic cancer cell lines. (a) Expression of COX-2 and MMP-9 was detected in pancreatic cell lines BxPC-3, Capan-1, PANC-1, and AsPC-1 by Western blotting. (b) PGE_2_ protein in the culture supernatant of BxPC-3 and Capan-1 cells was measured by Elisa. Data are expressed as mean ± SD, *n* = 3.

Western blotting was used to examine COX-2 and MMP-9 expression in pancreatic cell lines BxPC-3, Capan-1, PANC-1, and AsPC-1, and the examination showed COX-2 (72 kDa) and MMP-9 (92 kDa) expression varied in these cells. Both BxPC-3 and Capan-1 cells had strong expression of COX-2 and MMP-9, both of which, however, presented a weak expression in PANC-1 cells. No COX-2 expression was found in AsPC-1 cells (Figure 2). By Elisa we further revealed PGE_2_ protein in the culture supernatant in BxPC-3 and Capan-1 cells (Figure 2).

### 3.3. Inhibition of MMP-9 Expression by COX-2 Inhibitors

**Figure 3:**
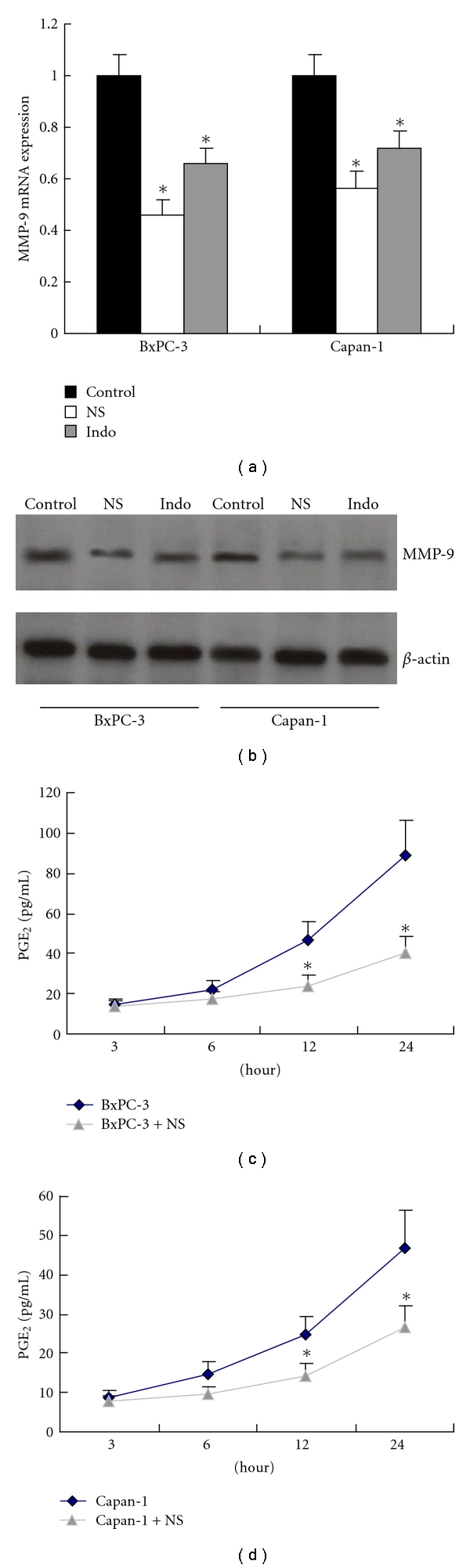
Inhibition of MMP-9 expression by NS398 or indomethacin (a) and (b) MMP-9 expression was inhibited in BxPC-3 and Capan-1 cells after treatment with NS398 (*P < 0.01, resp., versus control ) or indomethacin (*P < 0.01, resp., versus control). (c), (d) PGE_2_ protein levels were decreased in BxPC-3 and Capan-1 after treatment with NS398 for 12 hours (*P < 0.01, resp., versus control) or 24 hours (*P < 0.01, resp., versus control). Data are expressed as mean ± SD, *n* = 3. NS: NS398; indo: indomethacin.

In an attempt to explore the involvement of COX-2 in the upregulation of MMP-9, we treated particularly BxPC-3 and Capan-1 cells with selective COX-2 inhibitor NS398 (Sigma, 100 *μ*mol/L) for 24 hours, and we found MMP-9 expression was downregulated significantly in these cells (*P* < 0.01, resp., Figure 3). Similar results were observed when a nonselective COX-2 inhibitor indomethacin (Sigma, 100 mmol/L) was employed following the same procedure (*P* < 0.01, resp., Figure 3). In addition, it was found both BxPC-3 and Capan-1 secreted less PGE_2_ protein after treatment with NS398 for 12 hours (*P* < 0.01, resp.) or 24 hours (*P* < 0.01, resp., Figure 3).

### 3.4. Downregulation of MMP-9 by COX-2 siRNA

**Figure 4:**
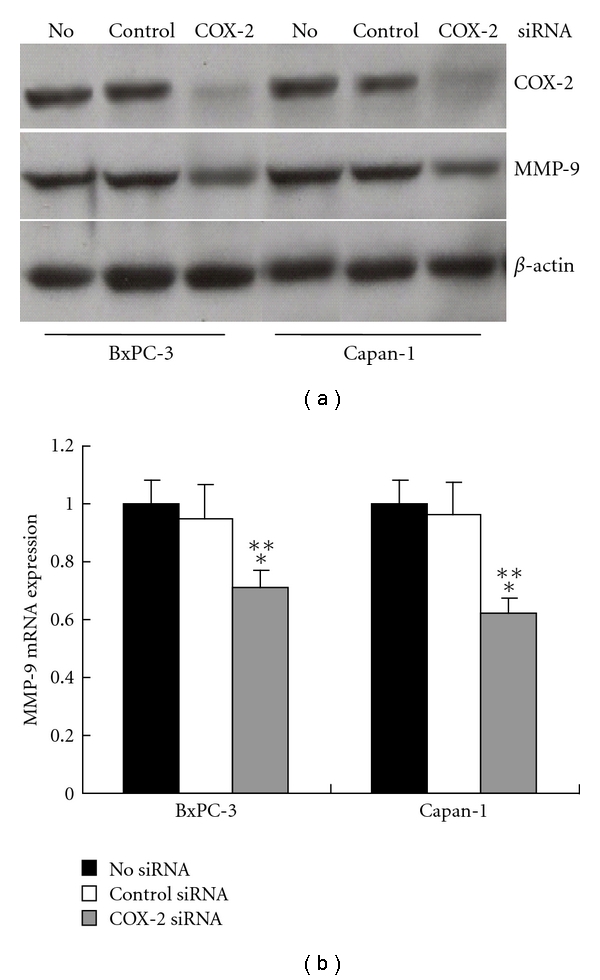
Downregulation of MMP-9 by COX-2 siRNA (a) Western blotting revealed expression of COX-2 was absent in BxPC-3 and Capan-1 after transfection with COX-2 siRNA plasmid. (b) After transfection with COX-2 siRNA plasmid, MMP-9 mRNA expression was downregulated significantly in BxPC-3 and Capan-1 cells, compared with cells with control siRNA (*P < 0.001, resp., versus control siRNA) or cells without siRNA (**P < 0.001, resp., versus no siRNA). Data are expressed as mean ± SD, *n* = 3.

COX-2 siRNA plasmid was used to transfect BxPC-3 and Capan-1 cells, and correspondingly nonsilencing siRNA plasmid was employed in the counterpart as control. It was observed that expression of COX-2 was absent in BxPC-3 and Capan-1 after transfection with COX-2 siRNA plasmid (Figure 4). Then expression of MMP-9 was detected by Western blot and real-time PCR, and it was noted in the detection that, after transfection with COX-2 siRNA plasmid, MMP-9 expression was downregulated significantly in BxPC-3 and Capan-1cells, compared with cells with control siRNA (*P* < 0.001, resp.) or cells without siRNA (*P* < 0.001, resp.) (Figure 4).

### 3.5. Involvement of COX-2 in the Upregulation of MMP-9 by TNF-*α* and LPS

**Figure 5:**
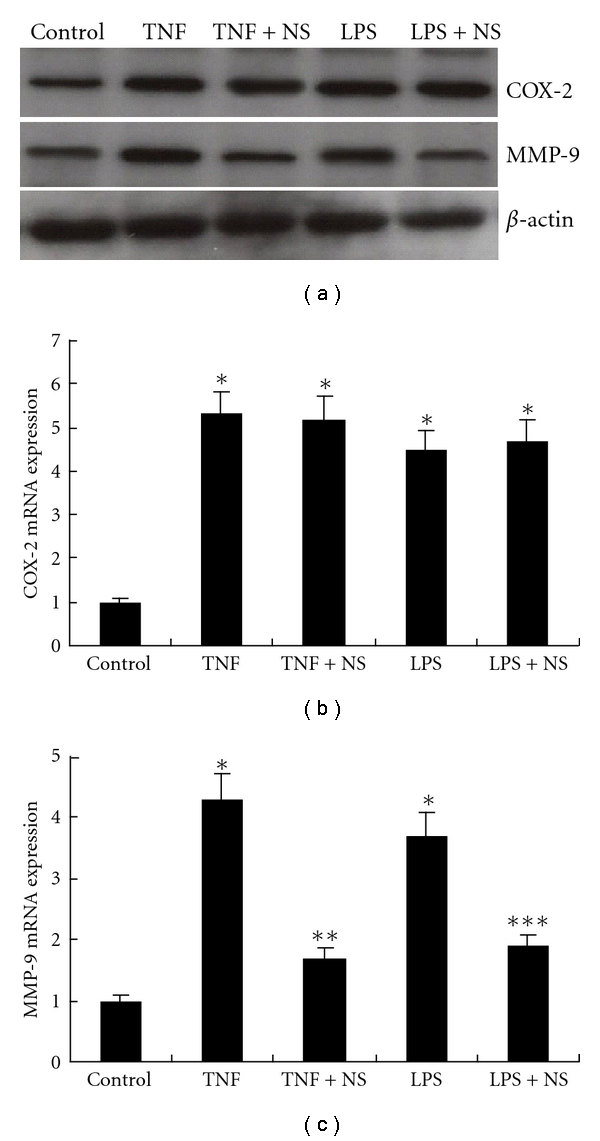
Involvement of COX-2 in the upregulation of MMP-9 by TNF-*α* and LPS. (a) Western blotting revealed expression of COX-2 and MMP-9 was increased in BxPC-3 after treatment with exogenous TNF-*α* or LPS. (b) COX-2 mRNA expression was upregulated by TNF-*α* or LPS (*P < 0.01, resp., versus control). NS398 had no effect on the upregulation. (c) MMP-9 mRNA expression was upregulated by TNF-*α* or LPS (*P < 0.01, resp., versus control). The upregulation was significantly inhibited by NS398. Data are expressed as mean ± SD, *n* = 3. NS: NS398.

BxPC-3 cells were treated with TNF-*α* (Sigma, 100 ng/mL) or LPS (Sigma, 100 ng/mL) for 6 hours in attempt to further explore the role of COX-2 in the upregulation of MMP-9 expression, and real time-PCR and Western blotting detection revealed that both COX-2 (*P* < 0.01, resp.) and MMP-9 (*P* < 0.01, resp.) were upregulated significantly (Figure 5). The upregulation of MMP-9 by TNF-*α* or LPS can be inhibited significantly by NS398 (*P* < 0.01, resp.), however, the upregulation of COX-2 cannot be inhibited by NS398 (Figure 5).

### 3.6. Upregulation of MMP-9 by Exogenous PGE_2_

**Figure 6:**
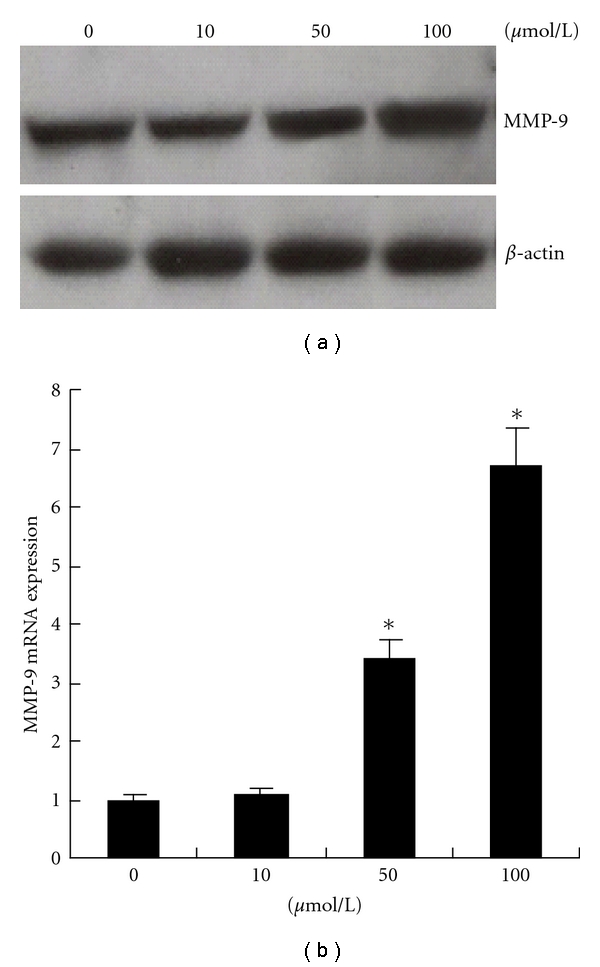
Upregulation of MMP-9 by exogenous PGE_2_ Western blotting (a) and real-time PCR (b) revealed MMP-9 was upregulated in BxPC-3 cells treated with 50 *μ*mol/L (*P < 0.01, versus 0 *μ*mol/L) or 100 *μ*mol/L (*P < 0.01, versus 0 *μ*mol/L). Data are expressed as mean ± SD, *n* = 3.

To explore whether PGE_2_ is involved in the upregulation of MMP-9 expression by COX-2, BxPC-3 cells were treated with 10, 50, or 100 *μ*mol/L PGE_2_ (Sigma) for 6 hours. Real-time PCR and Western blotting detection revealed MMP-9 was upregulated significantly in BxPC-3 cells treated with 50 *μ*mol/L (*P* < 0.01, Figure 6) or 100 *μ*mol/L (*P* < 0.01, Figure 6).

### 3.7. Migration Assays

**Figure 7:**
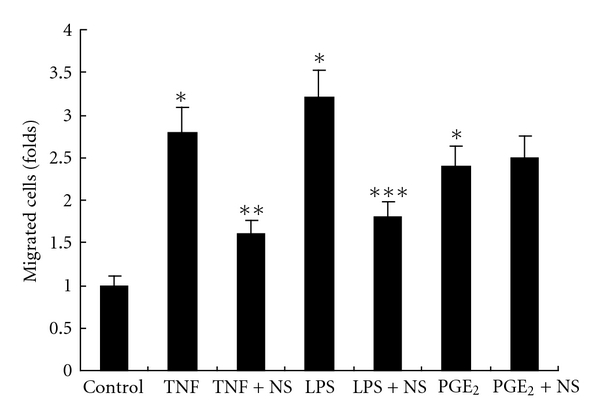
Migration analysis. Migration of BxPC-3 cells was increased after treatment with TNF-*α* (*P < 0.001, versus control), LPS (*P < 0.001, versus control), or PGE_2_ (*P < 0.001, versus control). The increase caused by TNF-*α* or LPS was inhibited remarkably when NS398 was added (**P < 0.01, versus TNF; ***P < 0.01, versus LPS). Data are expressed as mean ± SD, *n* = 3. NS: NS398.

Migration analysis was performed using the Matrigel invasion chamber in an attempt to investigate whether the upregulated MMP-9 was functional. There was a significant increase in the migration of BxPC-3 cells with TNF-*α* (*P* < 0.001), LPS (*P* < 0.001) or PGE_2_ (*P* < 0.001) treatment in the upper chamber, compared with control cells without treatment (Figure 7). Later on, however, the increase caused by TNF-*α* or LPS was inhibited remarkably when NS398 was added (*P* < 0.01, resp., Figure 7). The increase caused by PGE_2_ was unchanged.

## 4. Discussion

COX-2 shares catalytic activity and a 60% sequence homology with COX-1, another isoform of cyclooxygenase (COX). Though, unlike COX-1, COX-2 was not found to be expressed in most tissues in physiologic conditions, and it can be induced rapidly by some stimuli, such as LPS, cytokines, and growth factors [19, 20]. COX-2 and COX-1 catalyze the conversion of arachidonic acid to PGG_2_ and PGH_2_, which can subsequently be converted into PGE_2_ by PGE synthase. Under normal conditions, PGE_2_ is thought to be involved in some physiological functions including protection of gastric mucosa and regulation of glomerular filtration, whereas excessive production of PGE_2_ is responsible for some inflammatory diseases, such as rheumatoid arthritis and osteoarthritis. PGE_2_ in central nervous system plays a key role in fever, one of the most common signs of inflammatory diseases, and the decrease of its level by inhibition of COX-2 expression can lead to a suppression of the symptom. 

COX-2 and PGE_2_ are also blamed for their role in human cancer. Early evidence comes from studies on colorectal cancer, where their levels were found elevated, and accordingly, the COX-2 inhibitors helped to reduce the incidence [21, 22]. In pancreatic cancer, COX-2 was also reported to be overexpressed [14–16]. In contrast to the normal pancreatic tissues, where COX-2 expression was only found in islet cells, the cancer specimens show COX-2 expression was common but varied in our present studies. It was also found present in 3 of 4 pancreatic cancer cell lines, strong in BxPC-3 and Capan-1 cells, and weak in PANC-1 cells, but none of it was spotted in AsPC-1 cells. By Elisa we further revealed PGE_2_ protein in the culture supernatant in BxPC-3 and Capan-1 cells. These results suggest COX-2/PGE_2_ pathway may be involved in a part of the pancreatic cancer patients, though the mechanisms of COX-2 upregulation remain unclear. It is acknowledged that in inflammatory diseases, COX-2 is induced by LPS or some cytokines, such as IL-1*β* and TNF-*α* produced by some inflammatory cells, and therefore it could be reasonably speculated that in tumors, the upregulation of it may also be attributed to LPS and these cytokines. In fact, it was observed that LPS can stimulate some colon cancer cells to express COX-2 and release PGE_2_ by activation of NF-*κ*B [23]. In this study, we found both LPS and TNF-*α* increase COX-2 expression in pancreatic cancer cells, which suggests inflammatory microenvironment may be responsible for the overexpression of COX-2 in some pancreatic cancers.

In addition to cell proliferation and tumor growth, COX-2/PGE_2_ pathway is also involved in tumor invasiveness and metastasis [24–26]. It was observed that COX-2 expression increased invasiveness of colorectal cancers [27], and PGE_2_ boosted motility of colorectal cancer cells [28]. Furthermore, PGE_2_ was found to regulate COX-2-dependent invasion and metastasis of nonsmall cell lung cancer in an autocrine or paracrine manner [29]. In pancreatic cancer, one study has shown COX-2 expression was significantly associated with increased perineural invasion [18]. However, mechanisms of increased invasiveness and metastasis caused by COX-2/PGE_2_ are largely unknown. On the other hand, MMP-9, one of the two types IV collagenases, was extensively studied in human cancer, and a large body of evidence indicates its expression correlates well with tumor invasion and metastasis. In fact, its overexpression was observed in pancreatic cancers by several studies [30–32], and its levels were correlated positively with motility and invasiveness of pancreatic cancer cells. Consistent with these observations, our results indicated MMP-9 expression is a common phenomenon in pancreatic cancer, which can be regulated by a number of factors, including some inflammatory stimuli. Our study demonstrated that both LPS and TNF-*α* can upregulate it and increase the invasiveness of pancreatic cancer cells. Taken together, we speculated that COX-2/PGE_2_ may increase cell invasiveness and metastasis through MMP-9.

Our initial findings reveal a positive correlation of mRNA level of COX-2 expression with MMP-9 in pancreatic cancer specimens, and a probable connection between them in cancer cells. There was also strong expression of both agents in BxPC-3 and Capan-1 cells, but a weak one in PANC-1 cells. Second, MMP-9 expression was downregulated significantly in BxPC-3 and Capan-1 cells after treatment with COX-2 inhibitors or transfection with COX-2 siRNA plasmid to abrogate COX2 expression. Third, their expression was increased by pancreatic cancer cells due to exogenous TNF-*α* and LPS but the elevated expression of MMP-9 was inhibited by COX-2 inhibitor NS398 and was elevated again when treated with exogenous PGE_2_. Finally, there was a significant increase in the migration of BxPC-3 cells with TNF-*α*, LPS or PGE_2_ treatment, which, however, was inhibited remarkably by NS398. Taken together, all lines of evidence suggest COX-2/PGE_2_ pathway is involved in the upregulation of MMP-9 in pancreatic cancer.

COX-2 inhibitors have been shown to be effective in preventing colon cancer in animal models or clinical trials [33, 34]. Furthermore, a chronic usage of them, according to epidemiological studies, can decrease the incidence of colorectal cancer. The inhibitors were also found to suppress the development of pancreatic cancer in an animal model [35]. Such antitumor effects have been largely attributed to a consequent reduction in PGE_2_ levels, as one study showed, in cultured BxCP-3 cells, PGE_2_ levels were decreased significantly after treatment with indomethacin and NS398 (COX-2 inhibitors) [16]. Similar results were observed in our study, which revealed that COX-2 inhibitors, either selective or nonselective, decreased the PGE_2_ levels, reduced MMP-9 production, and inhibited cell invasiveness. On the other hand, exogenous PGE_2_ was found to increase MMP-9 expression and cell invasiveness. All these findings led to our conclusion that the effects of COX-2 inhibitors on pancreatic cancer cell invasiveness are primarily related to COX-2/PGE_2 _ pathway, though the possibility of other mechanisms' involvement can hardly be ruled out.

PGE_2_ in human cancer are not only blamed for cell growth and proliferation, but also for the invasion and metastasis. In fact, these effects are mediated by the particular corresponding G-protein-coupled receptors (GPCRs) of PGE_2_, named EP1, EP2, EP3, and EP4. Studies have shown only EP4 is implicated in cell invasion and migration. For instance, PGE_2_ was found to regulate COX-2-dependent invasion and metastasis of nonsmall cell lung cancer via the EP4 receptor signaling [29]. It is interesting that EP4 expression can also be regulated by LPS, just like PGE_2_. However, it still remains unknown whether EP4 mediates the action of PGE_2_ in pancreatic cancer, as its expression status was not determined in our study. Hopefully, it may offer another effective therapeutic target for these patients with further thorough laboratory observations and concrete findings.

In conclusion, we have determined COX-2/PGE_2_ pathway's involvement in the upregulation of MMP-9 in pancreatic cancer, and the restraint of COX-2 inhibitors on MMP-9 expression and cancer cell invasiveness. These results shed light on the connections between COX-2/PGE_2_ pathway with tumor growth, as well as invasiveness and metastasis in pancreatic cancer. With these important inflammatory mediators exhibiting complex effects in pancreatic cancer, a further safe conclusion is that there is a tight link between inflammation or inflammatory microenvironment with pancreatic carcinogenesis.
